# Supplementary material for: Extending resolution within a single imaging frame
Source: Nat Commun. 2022 Dec 2;13:7452. doi: 10.1038/s41467-022-34693-9 (PMC9718789; doi:10.1038/s41467-022-34693-9)
Supplement: Supplementary file 3 — Description of Additional Supplementary Files [file 41467_2022_34693_MOESM3_ESM.pdf]

Description of additional supplementary files

Movie S1. CRISPR/PAINT nanorulers resolved by MSSR. An image sequence of 100 frames of the CRISPR/PAINT nanoruler acquired by TIRF microscopy (diffraction-limited) was analyzed by sf-MSSR. Temporal analysis of t-MSSR data by PTF generates the super-resolved micrography. MSSR parameters: AMP = 20, FWHM of PSF for GattaPaint ATTO 550 = 3.31, order = 3.

Movie S2. Laser-written lithography pattern reconstruction at low SNR 5.4. This video shows the progressive gain in resolution image when one or several images are used for reconstruction in a low SNR set. The first image corresponds to a singleframe analysis with MSSR (no temporal analysis) while subsequent images result from the number of frames indicated on top. In this scenario, a single image is not sufficient for a reliable reconstruction, while at around 20 images used for temporal analysis the reconstruction recovers part of the expected shape of the fluorescent ring. However, there is a point where more images do not contribute to any further resolution enhancement, as seen in Supplementary Figure S28. sf-MSSR and t-MSSR parameters: AMP = 4, FWHM of PSF = 2.37, order = 1, PTF = TPM.

Movie S3. Laser-written lithography pattern reconstruction at high SNR 21.3. As in Supplementary Movie S2, this video shows the progressive gain in resolution but, in this case, a single image is sufficient for a reliable reconstruction. Further images used in the temporal analysis are not required. This is also quantified in Supplementary Figure S28. sf-MSSR and t-MSSR parameters: AMP = 4, FWHM of PSF = 2.37, order = 1, PTF = TPM.

Movie S4. Super-resolution sf-MSSR head. sf-MSSR extended-resolution single frame analysis of sperm acrosome exocytosis. As this process develops, a rise in FM4-64 fluorescence (magenta) and different dynamics in F-actin showed by SiR-actin (green) are seen. sf-MSSR parameters: AMP = 5, FWHM of PSF = 5, order = 0.

Movie S5. Super-resolution sf-MSSR fenestration. sf-MSSR extended-resolution single frame analysis of sperm membrane fenestration. Prior to AE, the plasma membrane is seen above the cortical F-actin. Once the AE occurs, the plasma membrane is fenestrated and the inner acrosomal membrane is now stained by FM4-64. As a result of this, F-actin localizes between membranes. sf-MSSR parameters: AMP = 5, FWHM of PSF = 5, order = 0.

Movie S6. Super-resolution t-MSSR head of 100 frames. t-MSSR super-resolution analysis of 100 temporal ‘pseudo’ replicates collected at the same scene of sperm acrosome exocytosis. As this process develops, a rise in FM4-64 fluorescence (magenta) and different dynamics in F-actin showed by SiR-actin (green) are seen. t-MSSR parameters: AMP = 5, FWHM of PSF = 5, order = 0, PTF = Mean.

Movie S7. Super-resolution t-MSSR fenestration of 100 frames. t-MSSR super-resolution analysis of 100 temporal ‘pseudo’ replicates collected at the same scene of sperm membrane fenestration. Prior to AE, the plasma membrane is seen above the cortical F-actin. Once the AE occurs, the plasma membrane is fenestrated and the inner acrosomal membrane is now stained by FM4-64. As a result of this, F-actin localizes between membranes. t-MSSR parameters: AMP = 5, FWHM of PSF = 5, order = 0, PTF = Mean.

Movie S8. Extended-resolution sf-MSSR fenestration of 100 frames at t0. sf-MSSR extended-resolution frame analysis of sperm membrane fenestration prior to AE. By using ‘pseudo’ replicates collected at the same scene before the AE occurs, different dynamics of plasma membrane and cortical F-actin are seen at a millisecond scale. sf-MSSR parameters: AMP = 5, FWHM of PSF = 5, order = 0.

Movie S9. Extended-resolution sf-MSSR fenestration of 100 frames at t13. sf-MSSR extended-resolution single frame analysis of sperm membrane fenestration after AE. By using ‘pseudo’ replicates collected at the same scene after the AE occurs, different dynamics of plasma membrane and cortical F-actin are seen at a millisecond scale. sf-MSSR parameters: AMP = 5, FWHM of PSF = 5, order = 0.

Movie S10. sf-MSSR video of live LLC-PK1 cells expressing mEmerald-EB3. Extended-resolution video of microtubule dynamics in live LLC-PK1 cells stably expressing mEmerald-EB3. Right side are two 2X enlargements of the areas depicted by the green squares. Time scale is 2 seconds per frame, playing at 30X speed. sf-MSSR parameters: AMP = 3, FWHM of PSF = 30, order = 0.

Movie S11. sf-MSSR video of an apoptotic LLC-PK1 cell expressing mEmerald-EB3. Extended-resolution video of microtubule dynamics in an apoptotic LLC-PK1 cell stably expressing mEmerald-EB3. Membrane blebbing can be seen in the contour of the cell. Time scale is 2 seconds per frame, playing at 30X speed. sf-MSSR parameters: AMP = 3, FWHM of PSF = 30, order = 0.

Movie S12. Extended-resolution 3D reconstruction of Z-stack slices of Arabidopsis thaliana root nucleosome. 3D representation of A. thaliana root nucleosomes labeled with RFP (p35s:H2B-RFP). The images were acquired with regular Olympus CLSM (see methods) and filtered with sf-MSSR. The 3D reconstruction was made by the 3D Viewer plugin of FIJI, with 86 slices of a Z-stack with a step size of 100 nm. For more details see methods.

Movie S13. DL reconstruction of epifluorescence BPAE cells for 2D and 3D images. Selective plane and rotated view of epifluorescence BPAE cells. Channels: mitochondria stained with MitoTrackerTM Red CMXRos (red), F-actin was stained withAlexa FluorTM 488 phalloidin (green), and the DNA of the nuclei was stained with DAPI (blue). The video was generated with Napari, with the plug-in animation: wizard.

Movie S14. sf-MSSR1 reconstruction of epifluorescence BPAE cells for 2D and 3D images. Selective plane and rotated view of sf-MSSR1 results of epifluorescence BPAE cells. Channels: mitochondria stained with MitoTrackerTM Red CMXRos (red), F-actin was stained with Alexa FluorTM 488 phalloidin (green), and the DNA of the nuclei was stained with DAPI (blue). sf-MSSR parameters: AMP = 10, FWHM of PSF = 2.77, order = 1. The video was generated with Napari, with the plug-in animation: wizard.

Description of the Supplementary Datasets:

Dataset 1: Imaging of the GATTA-SIM 140B nanoruler (GATTAquant DNA nanotechnologies) was carried out in an Elyra 7 microscope (Zeiss), equipped with a 60x 1.4 NA oil immersion objective and an extra 1.4x magnification lens. ZEN Black was used for image reconstruction, with parameters set to default.

Dataset 2: Using a Zeiss LSM880 inverted microscope with a Plan-Apochromat 63x/1.4 oil immersion objective, confocal images of the micropattern were acquired, with an effective pixel size of 44 nm. Excitation was provided by a 405 nm laser and the range of the collected fluorescence range was 420-480 nm. For the Airyscan detector, the same settings as in confocal imaging were applied for data collection. An optical filter BP 420-480 + LP 605 was used and image processing was done through the Airyscan algorithm (Zen Black, AIMApplication version 14.0.22.201) with a value of 6 for the 'strength' parameter.

Dataset 3: Mouse testes were removed from euthanized animals, followed by decapsulation and maceration in high-glucose MEM medium. Suspension was mixed thoroughly and left to settle; the supernatant was then collected and centrifuged at 7200 rpm for 1 min. The pellet was then resuspended in a 0.5 M sucrose solution and added to PFA-treated (1% in 0.015% Triton X-100) coverslides, which were incubated for at room temperature for 2 hours in a humidified environment. After incubation, slides were rinsed twice with a wetting agent solution (Kodak, 1464510) in water and allowed to air dry. SYCP3 was labeled with primary SCP-3 (D-1) antibody (Santa Cruz Biotechnology, SC-74569) and a secondary anti-mouse antibody fused with Alexa-568 (Thermo, A11004). Surface chromosome spreads were imaged in an Elyra 7 microscope (Zeiss), with a 60x 1.4 NA oil immersion objective and an a 1.4x magnification lens. Image reconstruction was performed in ZEN Black with parameters set to default. Experimental procedures were approved by the “Ministero della Salute" of Italy, authorization n.701/2018-PR.

Dataset 4: In either confocal or STED mode, immunofluorescence was performed as previously described in [1], using an upright Zeiss microscope with a mounted STEDYCON module and a Zeiss 100x 1.46 NA objective. Primary antibodies used were anti-H3K27ac (Active Motif, 39034) and anti-H3K27me3 (Abcam, ab6002), both at 1:200 dilution. Secondary anti-mouse and anti-rabbit antibodies used were labeled with STAR Red (Sigma-Aldrich, 52283) and STAR Orange (Sigma-Aldrich, 41367), respectively. Excitation was provided by 640 nm (@3%) and 775 nm (@96.5%) lasers for the STAR Red channel, whereas 561 nm (@7.8%) and 775 nm (@100%) lasers were used for the STAR Orange channel. Imaging of the samples was performed with 5 µs pixel dwell time, 64 µm pinhole aperture, 15-line accumulations and a pixel size of 20 nm. Experimental procedures were approved by the EMBL Rome Animal Facility in accordance with European and Italian

legislations. 1. Bošković, A., Bender, A., Gall, L., Ziegler-Birling, C., Beaujean, N., & Torres-Padilla, M.-E. Analysis of active chromatin modifications in early mammalian embryos reveals uncoupling of h2a.z acetylation and h3k36 trimethylation from embryonic genome activation. Epigenetics 7, 747–757 (2012).

Dataset 5: The GATTA-PAINT 40 RG nanoruler (GATTAquant, DNA nanotechnologies) consists of three fluorophores (ATTO 655/ATTO 542) separated 40 nm from each other. Imaging of the sample was performed on an Olympus IX-81 inverted microscope equipped with an Olympus UApo N 100X/1.49 NA, oil-immersion objective in TIRF illumination mode (Olympus, cellTIRF Illuminator) with ~200 nm evanescence field depth. Sample excitation was provided by 488 nm and 561 nm lasers at 23.1 mW laser power. 300 total images were collected with an EM-CCD sensor (Andor iXon 897), exposure time set to 50 ms per frame and a pixel size of 160 nm.

Dataset 6: The imaged PSFcheck pattern consists of a nanometric set of ring-like laser-written structures with a separation of 10 µm between each. The mean FWHM of these patterns was calculated to be 208 nm. A NanoImager-S microscope (ONI, Oxford Nanoimaging), equipped with a 100X 1.4 NA, oil-immersion objective (Olympus) was used to image the PSFcheck patterns in widefield fluorescence mode. Sample excitation was provided by a 561 nm laser and emission was collected with a 575-616.5 emission filter. A sCMOS sensor (Hamamatsu, Orca-0Flash4.0 V3) was used for image aquisition at 30 fps and 117 nm pixel size.

Dataset 7: Rotavirus cell infection and immunofluorescence. MA-104 Clone 1 cells (American Type Culture Collection; ATCC:CRL-2378.1; RRID:CVCL_3846) were cultured in DMEM-RS media supplemented with 5% fetal bovine serum at 37°C and 5% CO_2. Prior to infection, Rhesus rotavirus (RRV) was activated with trypsin (10 µg/ml) for 30 min at 37°C. MA104 cells grown on glass coverslips were infected with RRV at a multiplicity of infection (MOI) of 1. Cells were fixed and prepared for immunofluorescence after six hours post-infection. Using a STORM imaging buffer mounting medium (1.5% glucose oxidase + 100 mM β-mercaptoethanol), the coverslips were mounted onto the center of glass slides. Rotavirus replication machinery imaging. Cell infection micrographs were provided kindly by Garcés and collaborators [1]. Imaging was carried out with an Olympus IX-81 inverted microscope in TIRF mode (Olympus, cellTIRFM illuminator), with an evanescence field depth of 200 nm. The objective lens used was an Olympus UApo N 100x 1.4 NA oil-immersion, with an additional 1.6x magnification lens. Excitation of Alexa-488 and Alexa-568 was provided by 488 nm and 568 nm lasers, respectively. Using a laser-modulation protocol, previously described in [1], an EMCCD camera (iXon 897, Model No: DU-897E-CS0-#BV; Andor) was used to acquire the images. Acquisition rate was set to 20 fps with a pixel size of 100 nm. For processing with MSSR, the algorithm parameters were set as follows: AMP = 5, PSF = 3, Order = 1, GPU parallel computing = enabled, temporal analysis = Mean (100 frames). 1. Garcés S, Y. et al. Nanoscale organization of rotavirus replication machineries. elife 8, e42906, (2019).

Dataset 8: CD1 male mice (10 to 12 weeks old) were maintained in a 12-hour light and 12-hour dark cycle, at 23°C and 55±15% humidity, with water being always accessible. Animals were euthanized and cauda epididymal sperm collection followed. Cauda epididymis was cut at multiple sites, placed in 500 µl of non-capacitating medium (NC) and incubated at 37°C for 15 min. Supernatant was collected and pre-incubation with 100 nM SiR-actin in NC of recovered sperm took place for 10 min. Once dyed, sperm were once more incubated for at 37°C for 60 min in capacitating (CAP) conditions. NC used was a modified TYH medium (119.3 mM NaCl, 4.7 mM KCl, 1.71 mM $CaCl_2$•$2H_2O$, 1.2 mM KH_2PO_4, 1.2 mM $MgSO_4$•$7H_2O$, 0.51 mM sodium pyruvate, 5.56 mM glucose, 20 mM HEPES and 10 µg/ml gentamicin). For CAP conditions, 5 mg/ml BSA and 15 mM $NaHCO_3$ were added. Sperm were immobilized in coverslips treated with concanavalin-A (1 mg/ml). The imaging chamber was loaded with NC with 0.5 µM FM4-64 and 100 nM SiR-actin. Dye excitation was provided by 561 nm and 640 nm lasers. Using a NanoImager-S microscope (ONI, Oxford Nanoimaging Ltd) equipped with a 100X, 1.4 NA, oil-immersion objective (Olympus), 100 frames were acquired every 0.5 min for a total of 20 min, with a pixel size of 117 nm. Experimental procedures were approved by the Bioethics Committee of the Biotechnology Institute of the National Autonomous University of Mexico.

Dataset 9: *Arabidopsis thaliana* seeds were surface sterilized, germinated and grown in Murashigue and Skoog medium at pH 5.7 and supplemented with vitamins (0.1 mg l-1 pyridoxine, 0.1 mg l-1 nicotinic acid), 0.8% agar, and 1% sucrose. Plants were grown at 21°C, 16/8-hour light/dark periods at 105 $\mu mol/m^2s^2$ light intensity. Using a Zeiss Axiovert 200M microscope and a C-APO 63X, 1.2NA objective (Oberkochen, Germany), confocal volumetric imaging of the plant material was performed, with a pixel size of 404 nm and Z step size of 500 nm. Excitation of the sample was provided by a 488 nm laser, and a filter cube with 525/45 nm and 630/92 nm bandpass filters was used for yellow and red emission light collection, respectively. An inverted Olympus FV1000-IX81 confocal microscope equipped with a LUMFLN×60, 1.3N A S objective was used for root cell nuclear imaging. Sample excitation was achieved with a 543 nm laser and a BA560-660 filter was used for emission light collection. Pixel size was 41 nm, with a Z step size of 100 nm. A custom-built selective plane illumination microscopy (SPIM) system was used for imaging of primary root cells expressing p35s:H2B-R-RF. Sample excitation was achieved with a 561 nm laser using stroboscopic illumination. Emission was filtered via a multi-bandpass emission filter (Semrock, FF01-446/523/600/677-25 BrightLine). Volumetric imaging was done by mounting the sample on a four dimensional (XYZ, and Y rotation) motorized stage (Picard Industries). A sCMOS sensor (Hamamatsu, ORCA-Flash4.0 V2) was used for signal recording. The OpenSPIM plugin of µmanager (v.1.4 for windows) was used for control of acquisition parameters, sample translation and stroboscopic illumination. Collected images had a pixel size of 0.325µm, Z step size of 100 nm and a Y rotation step of 1.8°. Experimental procedures were

approved by the Bioethics Committee of the Biotechnology Institute of the National Autonomous University of Mexico.

Dataset 10: Using a custom code written in R, an image series of EM-CCD sensor noise was simulated, based on the equations and theory provided by Hirsch and collaborators [1]. 1. M. Hirsch, R. J. Wareham, M. L. Martin-Fernandez, M. P. Hobson, and D. J. Rolfe, "A stochastic model for electron multiplication charge-coupled devices – from theory to practice," PLOS ONE 8, 1–13 (2013).

Dataset 11: Stable LLC-PK1 cell line (ATCC:CL-101) was generated and provided by Michael W. Davidson [1,2]. Using a Zeiss Celldiscoverer 7 microscope with a 100X/1.47 NA oil-immersion objective (Plan-Apochromat, Zeiss) and a sCMOS sensor (Hamamatsu, ORCA-Fusion, C15440-20UP), cultured LLC-PK1 cells expressing mEmerald-EB3 were imaged with a 43 nm pixel size. Exposure time was 100 ms, with each frame being captured every two seconds. During acquisition, temperature and CO2 control was set to 37°C and 5%, respectively. mEmerald-EB3 was excited using a 488 nm laser at 1% power, with a FITC filter for fluorescence collection. Acquisition software ZEN 3.2 (blue edition) was used for the imaging protocol. 1. Rizzo, M. A., Davidson, M. W., & Piston, D. W. (2009). Fluorescent protein tracking and detection: fluorescent protein structure and color variants. Cold Spring Harbor Protocols, 2009(12), pdb-top63. 2. Huang, F., Hartwich, T., Rivera-Molina, F. et al. Video-rate nanoscopy using sCMOS camera–specific single-molecule localization algorithms. Nat Methods 10, 653–658 (2013).

Dataset 12: A self-assembling polypeptide (C-S_{10}-B, labeled in green) binds to a DNA that has been pre-decorated with CRISPR-dCas12a (labeled in magenta) via multiple CRISPR-RNAs (crRNAs). C-S_{10}-B binds and diffuses along DNA but cannot move beyond stably bound dCas12a. During self-assembly, large clusters of C-S_{10}-B commonly form and colocalize with dCas12a. Bacteriophage λ DNA (λ DNA) (NEB, N3011S) was mixed in T4 DNA ligase (NEB, M0202S) reaction buffer with biotinylated oligos complementary to λDNA cohesive ends, for 15 min at 70°C, followed by a cool down to 15 °C for over 2h Ligation took place overnight at room temperature. After T4 DNA ligase inactivation with 2 M NaCl, the biotinylated DNA was purified on a Sephacryl S-1000 size exclusion column (GE Healthcare). Using a lipid solution (1.954% DOPC, 0.04% DOPE-mPEG2k and 0.006% DOPE-biotin) in buffer (10 mM Tris-HCL pH 8, 100 mM NaCl), the flowcell was passivated at room temperature for 30 min. Next, BSA buffer (40 mM Tris-HCl pH 8, 2 mM $MgCl_2$, 0.2 mg/mL BSA) was used to wash the flowcell, followed by incubation for 10 min. BSA buffer containing biotinylated DNA was injected into the flowcell, before washing out all the non-tethered DNA material. Imaging was done in BSA buffer supplemented with 100 mM NaCl, 5 mM $MgCl_2$, 2 mM DTT. dCas12a was mixed with the crRNA pool at a 1:10 molar ratio in buffer (20 mM Tris-HCl pH 8.0, 100 mM NaCl, 5 mM $MgCl_2$, 2% glycerol, 2 mM DTT) for 30 min at 37°C. The formed ribonucleoprote in particle complexes (10 nM) were injected into the flowcell to induce DNA binding

for 30 min at room temperature. Monoclonal ANTI-FLAG ® BioM2-Biotin (Sigma-Aldrich, F9291) conjuga ted to quantum dots (Thermo, Q21361MP) were used to label dCas12a. C-S_{10}-B was labeled at a single N-terminal cysteine with maleimide-Alexa-488. Imaging was carried out in an inverted Nikon Ti-E micros cope at 60X magnification. Excitation of the sample was provided by a 488 nm laser. Emission light was split with a 638 nm dichroic beam splitter (Chroma) and registered by two EM-CCD cameras (Andor iXon DU897). Image processing was done in FIJI.

Dataset 13: Using the Nanoimager-S microscope (ONI, Oxford Nanoimaging) with a sCMOS sensor (Hamamatsu, ORCA-Flash4.0 V2), FluoCells^{TM} Prepared Slide #1 (Thermo, #F36924) were imaged with a 100X, 1.4 NA, oil-immersion objective (Olympus). DAPI, Alexa-488 and MitoTracker^{TM} Red excitation was delivered by 405 nm, 473 nm and 561 nm lasers, respectively. Light was collected while using two emission filters (1: 525/50; 2: Band 1 575-616.5) and a Channel Splitter dichroic 561 LP. A 3D Z-stack of the sample was acquired for each channel. 22 frames were generated, each separated 50 nm from each other in Z.
