# Supplementary figures and images for: Extending resolution within a single imaging frame
Source: Nat Commun. 2022 Dec 2;13:7452. doi: 10.1038/s41467-022-34693-9 (PMC9718789; doi:10.1038/s41467-022-34693-9)

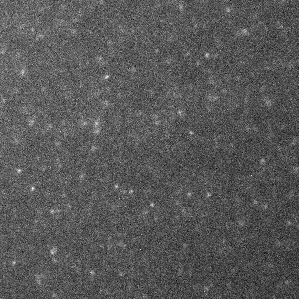

Supplement: Supplementary file 4 — Dataset 1 [file 41467_2022_34693_MOESM4_ESM.zip › DL Gatta-SIM nanoruler full FOV.tif]

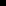

Supplement: Supplementary file 4 — Dataset 1 [file 41467_2022_34693_MOESM4_ESM.zip › DL Gatta-SIM nanoruler ROI-1.tif]

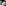

Supplement: Supplementary file 4 — Dataset 1 [file 41467_2022_34693_MOESM4_ESM.zip › DL Gatta-SIM nanoruler ROI-2.tif]

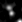

Supplement: Supplementary file 4 — Dataset 1 [file 41467_2022_34693_MOESM4_ESM.zip › SIM Gatta-SIM nanoruler ROI-1.tif]

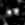

Supplement: Supplementary file 4 — Dataset 1 [file 41467_2022_34693_MOESM4_ESM.zip › SIM Gatta-SIM nanoruler ROI-2.tif]

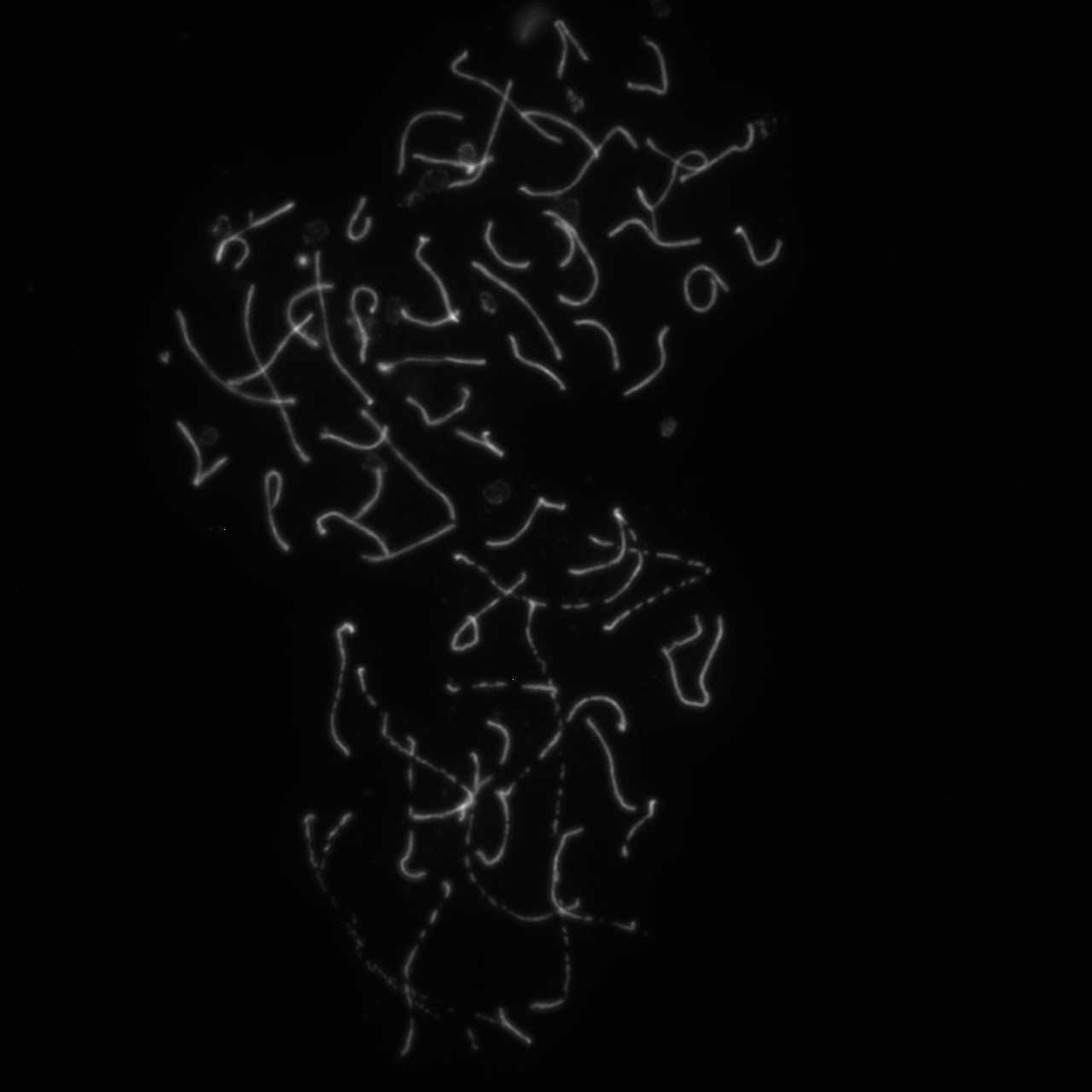

Supplement: Supplementary file 6 — Dataset 3 [file 41467_2022_34693_MOESM6_ESM.zip › DL Synapsed homologs of meiotic mouse chromosomes full FOV.tif]

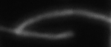

Supplement: Supplementary file 6 — Dataset 3 [file 41467_2022_34693_MOESM6_ESM.zip › DL Synapsed homologs of meiotic mouse chromosomes ROI-1.tif]

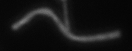

Supplement: Supplementary file 6 — Dataset 3 [file 41467_2022_34693_MOESM6_ESM.zip › DL Synapsed homologs of meiotic mouse chromosomes ROI-2.tif]

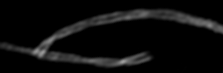

Supplement: Supplementary file 6 — Dataset 3 [file 41467_2022_34693_MOESM6_ESM.zip › SIM Synapsed homologs of meiotic mouse chromosomes ROI-1.tif]

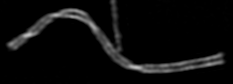

Supplement: Supplementary file 6 — Dataset 3 [file 41467_2022_34693_MOESM6_ESM.zip › SIM Synapsed homologs of meiotic mouse chromosomes ROI-2.tif]

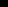

Supplement: Supplementary file 8 — Dataset 5 [file 41467_2022_34693_MOESM8_ESM.zip › DL GATTA-PAINT 100 nm nanoruler series.tif]

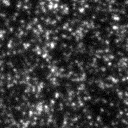

Supplement: Supplementary file 8 — Dataset 5 [file 41467_2022_34693_MOESM8_ESM.zip › DL GATTA-PAINT 40 nm nanoruler ATTO-488.tif]

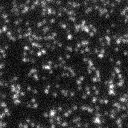

Supplement: Supplementary file 8 — Dataset 5 [file 41467_2022_34693_MOESM8_ESM.zip › DL GATTA-PAINT 40 nm nanoruler ATTO-550.tif]

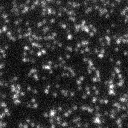

Supplement: Supplementary file 8 — Dataset 5 [file 41467_2022_34693_MOESM8_ESM.zip › DL GATTA-PAINT 40 nm nanoruler ATTO-655.tif]

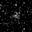

Supplement: Supplementary file 9 — Dataset 6 [file 41467_2022_34693_MOESM9_ESM.zip › ACsN-corrected PSFcheck pattern/ACsN-PSFcheck_ring_pattern_SNR=2.2.tif]

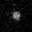

Supplement: Supplementary file 9 — Dataset 6 [file 41467_2022_34693_MOESM9_ESM.zip › ACsN-corrected PSFcheck pattern/ACsN-PSFcheck_ring_pattern_SNR=5.4.tif]

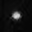

Supplement: Supplementary file 9 — Dataset 6 [file 41467_2022_34693_MOESM9_ESM.zip › ACsN-corrected PSFcheck pattern/ACsN-PSFcheck_ring_pattern_SNR=8.2.tif]

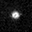

Supplement: Supplementary file 9 — Dataset 6 [file 41467_2022_34693_MOESM9_ESM.zip › Photons PSFcheck pattern/Photons-PSFcheck_ring_pattern_SNR=10.4.tif]

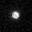

Supplement: Supplementary file 9 — Dataset 6 [file 41467_2022_34693_MOESM9_ESM.zip › Photons PSFcheck pattern/Photons-PSFcheck_ring_pattern_SNR=15.07.tif]

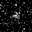

Supplement: Supplementary file 9 — Dataset 6 [file 41467_2022_34693_MOESM9_ESM.zip › Photons PSFcheck pattern/Photons-PSFcheck_ring_pattern_SNR=2.2.tif]

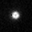

Supplement: Supplementary file 9 — Dataset 6 [file 41467_2022_34693_MOESM9_ESM.zip › Photons PSFcheck pattern/Photons-PSFcheck_ring_pattern_SNR=21.3.tif]

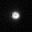

Supplement: Supplementary file 9 — Dataset 6 [file 41467_2022_34693_MOESM9_ESM.zip › Photons PSFcheck pattern/Photons-PSFcheck_ring_pattern_SNR=29.4.tif]

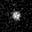

Supplement: Supplementary file 9 — Dataset 6 [file 41467_2022_34693_MOESM9_ESM.zip › Photons PSFcheck pattern/Photons-PSFcheck_ring_pattern_SNR=5.4.tif]

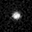

Supplement: Supplementary file 9 — Dataset 6 [file 41467_2022_34693_MOESM9_ESM.zip › Photons PSFcheck pattern/Photons-PSFcheck_ring_pattern_SNR=8.2.tif]

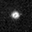

Supplement: Supplementary file 9 — Dataset 6 [file 41467_2022_34693_MOESM9_ESM.zip › Raw PSFcheck ring pattern/PSFcheck_ring_pattern_SNR=10.4.tif]

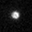

Supplement: Supplementary file 9 — Dataset 6 [file 41467_2022_34693_MOESM9_ESM.zip › Raw PSFcheck ring pattern/PSFcheck_ring_pattern_SNR=15.07.tif]

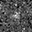

Supplement: Supplementary file 9 — Dataset 6 [file 41467_2022_34693_MOESM9_ESM.zip › Raw PSFcheck ring pattern/PSFcheck_ring_pattern_SNR=2.2.tif]

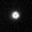

Supplement: Supplementary file 9 — Dataset 6 [file 41467_2022_34693_MOESM9_ESM.zip › Raw PSFcheck ring pattern/PSFcheck_ring_pattern_SNR=21.3.tif]

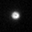

Supplement: Supplementary file 9 — Dataset 6 [file 41467_2022_34693_MOESM9_ESM.zip › Raw PSFcheck ring pattern/PSFcheck_ring_pattern_SNR=29.4.tif]

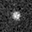

Supplement: Supplementary file 9 — Dataset 6 [file 41467_2022_34693_MOESM9_ESM.zip › Raw PSFcheck ring pattern/PSFcheck_ring_pattern_SNR=5.5.tif]

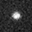

Supplement: Supplementary file 9 — Dataset 6 [file 41467_2022_34693_MOESM9_ESM.zip › Raw PSFcheck ring pattern/PSFcheck_ring_pattern_SNR=8.2.tif]

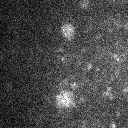

Supplement: Supplementary file 10 — Dataset 7 [file 41467_2022_34693_MOESM10_ESM.zip › NSP2-red_NSP4-green.tif]

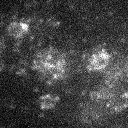

Supplement: Supplementary file 10 — Dataset 7 [file 41467_2022_34693_MOESM10_ESM.zip › NSP2-red_NSP5-green.tif]

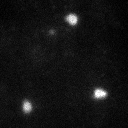

Supplement: Supplementary file 10 — Dataset 7 [file 41467_2022_34693_MOESM10_ESM.zip › NSP2-red_VP4-green.tif]

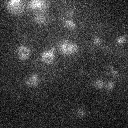

Supplement: Supplementary file 10 — Dataset 7 [file 41467_2022_34693_MOESM10_ESM.zip › NSP2-red_VP7-green_1.tif]

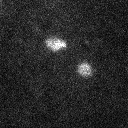

Supplement: Supplementary file 10 — Dataset 7 [file 41467_2022_34693_MOESM10_ESM.zip › NSP2-red_VP7-green_2.tif]

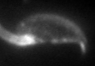

Supplement: Supplementary file 11 — Dataset 8 [file 41467_2022_34693_MOESM11_ESM.zip › Sperm Head Acrosome Reaction induction.tif]

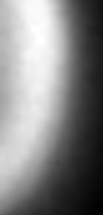

Supplement: Supplementary file 11 — Dataset 8 [file 41467_2022_34693_MOESM11_ESM.zip › Sperm Head Fenestration site post-induction.tif]

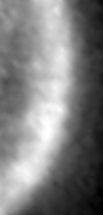

Supplement: Supplementary file 11 — Dataset 8 [file 41467_2022_34693_MOESM11_ESM.zip › Sperm Head Fenestration site pre-induction.tif]

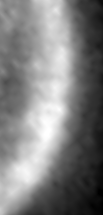

Supplement: Supplementary file 11 — Dataset 8 [file 41467_2022_34693_MOESM11_ESM.zip › Sperm Head Fenestration site.tif]

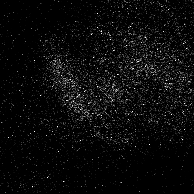

Supplement: Supplementary file 12 — Dataset 9 [file 41467_2022_34693_MOESM12_ESM.zip › 3D Z-stack of Arabidopsis thaliana root nucleosome.tif]

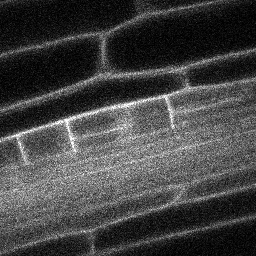

Supplement: Supplementary file 12 — Dataset 9 [file 41467_2022_34693_MOESM12_ESM.zip › Confocal image series of an early-stage Arabidopsis thaliana lateral root primordium.tif]

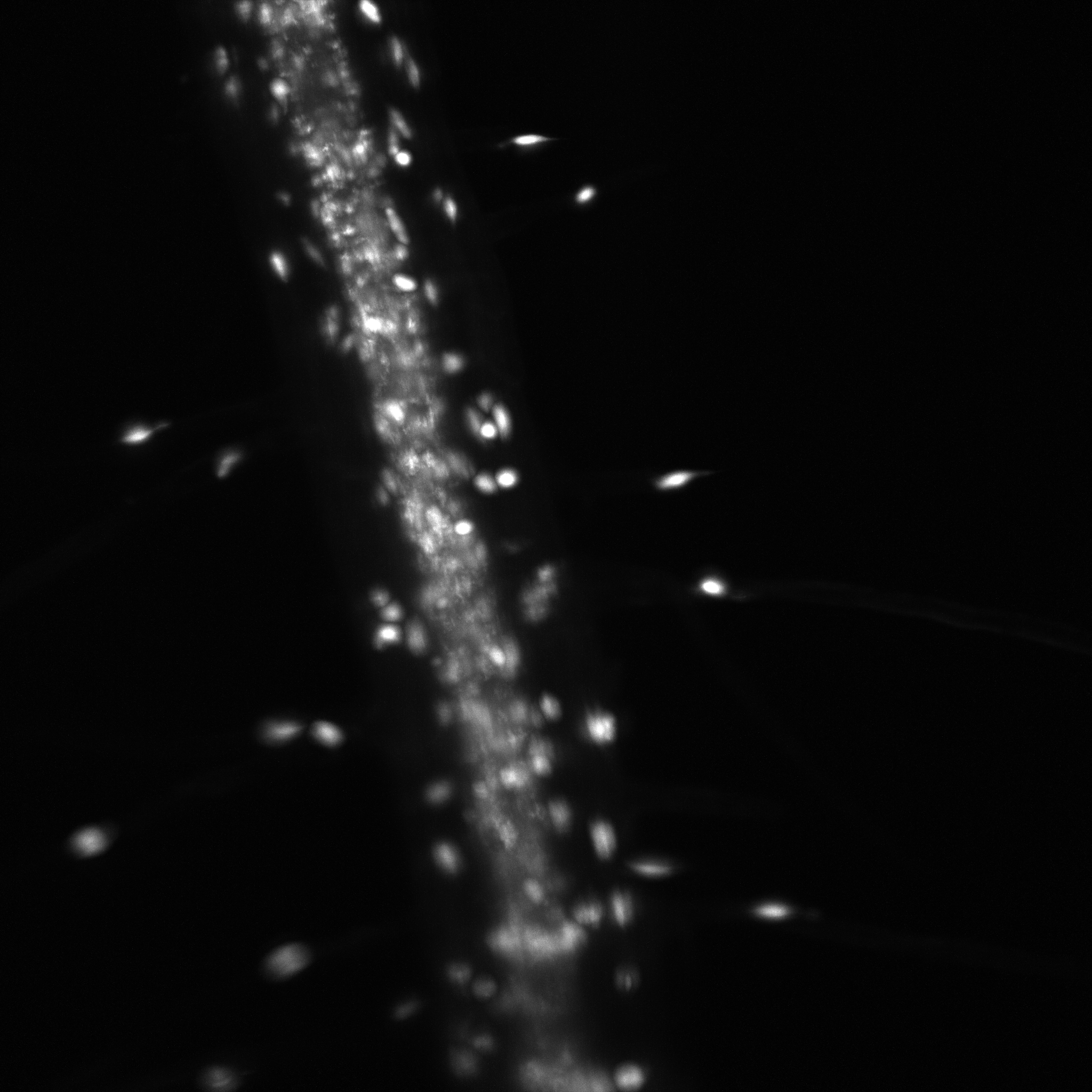

Supplement: Supplementary file 12 — Dataset 9 [file 41467_2022_34693_MOESM12_ESM.zip › SPIM nuclei imaging of Arabidopsis thaliana root cells.tif]

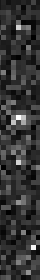

Supplement: Supplementary file 15 — Dataset 12 [file 41467_2022_34693_MOESM15_ESM.zip › DNA molecule with bound C-S_10-B and dCas12a.tif]

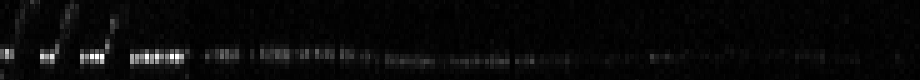

Supplement: Supplementary file 15 — Dataset 12 [file 41467_2022_34693_MOESM15_ESM.zip › Kymograph dCas12a.tif]

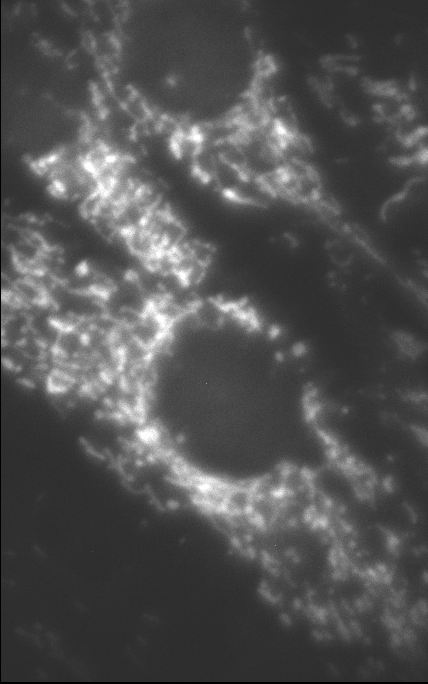

Supplement: Supplementary file 16 — Dataset 13 [file 41467_2022_34693_MOESM16_ESM.zip › Volumetric imaging of fluorescently labeled BPAE cells.tif]
