# Supplementary material for: Extending resolution within a single imaging frame
Source: Nat Commun. 2022 Dec 2;13:7452. doi: 10.1038/s41467-022-34693-9 (PMC9718789; doi:10.1038/s41467-022-34693-9)
Supplement: Supplementary file 31 — Reporting Summary [file 41467_2022_34693_MOESM31_ESM.pdf]

# nature portfolio

Corresponding author(s): Adán Guerrero

Last updated by author(s): Aug 3, 2022

# Reporting Summary

Nature Portfolio wishes to improve the reproducibility of the work that we publish. This form provides structure for consistency and transparency in reporting. For further information on Nature Portfolio policies, see our Editorial Policies and the Editorial Policy Checklist.

## Statistics

For all statistical analyses, confirm that the following items are present in the figure legend, table legend, main text, or Methods section.

| n/a | Confirmed | |
|---|---|---|
| [ ] | [x] | The exact sample size (*n*) for each experimental group/condition, given as a discrete number and unit of measurement |
| [x] | [ ] | A statement on whether measurements were taken from distinct samples or whether the same sample was measured repeatedly |
| [x] | [ ] | The statistical test(s) used AND whether they are one- or two-sided<br>*Only common tests should be described solely by name; describe more complex techniques in the Methods section.* |
| [x] | [ ] | A description of all covariates tested |
| [x] | [ ] | A description of any assumptions or corrections, such as tests of normality and adjustment for multiple comparisons |
| [ ] | [x] | A full description of the statistical parameters including central tendency (e.g. means) or other basic estimates (e.g. regression coefficient) AND variation (e.g. standard deviation) or associated estimates of uncertainty (e.g. confidence intervals) |
| [x] | [ ] | For null hypothesis testing, the test statistic (e.g. *F*, *t*, *r*) with confidence intervals, effect sizes, degrees of freedom and *P* value noted<br>*Give P values as exact values whenever suitable.* |
| [x] | [ ] | For Bayesian analysis, information on the choice of priors and Markov chain Monte Carlo settings |
| [x] | [ ] | For hierarchical and complex designs, identification of the appropriate level for tests and full reporting of outcomes |
| [ ] | [x] | Estimates of effect sizes (e.g. Cohen's *d*, Pearson's *r*), indicating how they were calculated |

*Our web collection on statistics for biologists contains articles on many of the points above.*

## Software and code

Policy information about availability of computer code

| | |
|---|---|
| Data collection | NanoScope Analysis Software v1.89<br>Airyscan algorithm (Zen Black, AIMApplication version 14.0.22.201)<br>OpenSPIM plugin 64-Bits of µmanager (v.1.4 for windows)<br>ZEN 3.2 (blue edition) |
| Data analysis | MSSR (v. 2.0.0) source code for R, python, MATLAB and FIJI/ImageJ platforms is available at https://github.com/MSSRSupport/MSSR.<br>MATLAB (v. 2019b, 2020a and 2021a)<br>R (v. 4.1.1)<br>Java (v. 8)<br>ImageJ (v. 1.53c)<br>Napari (Alpha build)<br>NimOS (v. 1.19)<br>MicroManager (Micro-Manager v. 1.4.21)<br>TrackMate (v 7.6.1)<br>Python (v. 3.8.0)<br>Icy Icy (v. 2.4)<br>DaVinci Resolve (v. 17.4.2)<br>ACsN (last version updated Nov 18, 2021 - doi: 10.1038/s41467-019-13841-8)<br>SRRF (last version updated Nov 27, 2020 - doi: 10.1038/ncomms12471) |

MUSICAL (ImageJ MusiJ plugin v. 0.94, MatLab v. 1.0 - doi: 10.1038/ncomms13752)
ESI (last version updated May 25, 2016 - doi: 10.1021/acsphotonics.5b00307)

For manuscripts utilizing custom algorithms or software that are central to the research but not yet described in published literature, software must be made available to editors and reviewers. We strongly encourage code deposition in a community repository (e.g. GitHub). See the Nature Portfolio guidelines for submitting code & software for further information.

## Data

Policy information about availability of data

All manuscripts must include a data availability statement. This statement should provide the following information, where applicable:

- Accession codes, unique identifiers, or web links for publicly available datasets
- A description of any restrictions on data availability
- For clinical datasets or third party data, please ensure that the statement adheres to our policy

All raw data used or generated in this study have been deposited in the Zenodo OpenAIRE database and are available under unique accession codes, located in the Methods section of this manuscript. Source data are provided with this paper. The source code used to build the plots within this paper are available from the corresponding author upon reasonable request. DOI for each source data are also provided here:
a. Gatta-SIM nanorulers. DOI: 10.5281/zenodo.6941792
b. Airyscan and Confocal line pattern. DOI: 10.5281/zenodo.6848342
c. Synapsed homologs of meiotic mouse chromosomes visualized by TIRFM. DOI: 10.5281/zenodo.6865142
d. STED immunofluorescence imaging of histone protein H3K27 in a 2-cell stage mice embryo. DOI: 10.5281/zenodo.6865168
e. CRISPR-PAINT nanorulers. DOI: 10.5281/zenodo.6850637
f. PSFcheck ring pattern at various SNR. DOI: 10.5281/zenodo.6955019
g. Rotavirus viroplasms. DOI: 10.5281/zenodo.6850357
h. Mouse sperm acrosome exocytosis. DOI: 10.5281/zenodo.6850232
i. Volumetric imaging of Arabidopsis thaliana root cells. DOI: 10.5281/zenodo.6850745
j. EM-CCD noise image sequence. DOI: 10.5281/zenodo.6955070
k. Live-cell imaging of LLC-PK1 cells microtubule dynamics. DOI: 10.5281/zenodo.6850280
l. DNA curtain assay for dCas12a/CS10B colocalization. DOI: 10.5281/zenodo.6865120
m. Volumetric imaging of fluorescently labeled BPAE cells. DOI: 10.5281/zenodo.6865066

## Human research participants

Policy information about studies involving human research participants and Sex and Gender in Research.

| | |
|---|---|
| Reporting on sex and gender | No human research participants were used in this study. |
| Population characteristics | N/A |
| Recruitment | N/A |
| Ethics oversight | N/A |

Note that full information on the approval of the study protocol must also be provided in the manuscript.

# Field-specific reporting

Please select the one below that is the best fit for your research. If you are not sure, read the appropriate sections before making your selection.

[x] Life sciences  [ ] Behavioural & social sciences  [ ] Ecological, evolutionary & environmental sciences

For a reference copy of the document with all sections, see nature.com/documents/nr-reporting-summary-flat.pdf

# Life sciences study design

All studies must disclose on these points even when the disclosure is negative.

| | |
|---|---|
| Sample size | No sample size calculation was performed. A portion of the manuscript is dedicated to data simulation and numerical approximations to test the MSSR algorithm. These demonstrations do not require sample size calculation as the analytical approach is deterministic. The biological examples provided aim to demonstrate the ability of MSSR to extend spatial resolution in a wide range of experimental scenarios. However, these examples were generated from previously published data, hence sample size calculation nor large sample sizes are required. |
| Data exclusions | For Figure 7 in the main manuscript, CRISPR/dCas12a nanoruler binding site distances were computed by means of scanning all the individual super-resolved emitters in close proximity. Given that these binding sites are theoretically separated 100 nm from each other along the dsDNA, distances greater than 350 nm were excluded from the analysis. |

| | |
|---|---|
| Replication | Since MSSR is a deterministic approach for image processing, applying the algorithm over the same data set always provides the same result. |
| Randomization | Sample randomization was not relevant to this study. The aim of this work is to test the capabilities of MSSR as an image processing tool for spatial resolution extension, rather than interpretation of biological data and/or study of organism behavior. |
| Blinding | Blinding was not relevant for data collection. The aim of this work is to test the capabilities of MSSR as an image processing tool for spatial resolution extension, rather than interpretation of biological data and/or study of organism behavior. |

# Reporting for specific materials, systems and methods

We require information from authors about some types of materials, experimental systems and methods used in many studies. Here, indicate whether each material, system or method listed is relevant to your study. If you are not sure if a list item applies to your research, read the appropriate section before selecting a response.

## Materials & experimental systems

| n/a | Involved in the study | |
|---|---|---|
| ☐ | ☒ | Antibodies |
| ☐ | ☒ | Eukaryotic cell lines |
| ☒ | ☐ | Palaeontology and archaeology |
| ☐ | ☒ | Animals and other organisms |
| ☒ | ☐ | Clinical data |
| ☒ | ☐ | Dual use research of concern |

## Methods

| n/a | Involved in the study | |
|---|---|---|
| ☒ | ☐ | ChIP-seq |
| ☒ | ☐ | Flow cytometry |
| ☒ | ☐ | MRI-based neuroimaging |

## Antibodies

| | |
|---|---|
| Antibodies used | Mouse monoclonal antibody VP4 (2G4) (Harry B. Greenberg, Stanford University. PMID:2431540). Dilution 1:1000.<br>Mouse monoclonal antibody VP7 (M60) (Harry B. Greenberg, Stanford University. PMID:2431540). Dilution 1:2000.<br>Mouse monoclonal antibody VP7 (159) (Harry B. Greenberg, Stanford University. PMID:2431540). Dilution 1:2000.<br>Mouse polyclonal antibody NSP2 (Made by our laboratory, PMID: 9645203; RRID:AB_2802096). Dilution 1:100.<br>Rabbit polyclonal antibody NSP2 (Made by our laboratory, PMID: 9645203; RRID:AB_2802097). Dilution 1:2000.<br>Rabbit polyclonal antibody NSP4 (Made by our laboratory, PMID: 18385250; RRID:AB_2802094). Dilution 1:1000.<br>Rabbit polyclonal antibody NSP5 (Made by our laboratory, PMID:9645203; RRID:AB_2802098). Dilution 1:2000.<br>Goat anti-rabbit Alexa 568 (Invitrogen, A-11011). Dilution 1:10000.<br>Goat anti-mouse Alexa 488 (Invitrogen, A-10680). Dilution 1:10000.<br>Primary mouse anti-H3K27me (Abcam, ab6002). Dilution 1:200.<br>Primary rabbit anti-H3K27ac (Active Motif, 39034). Dilution 1:200.<br>Secondary goat anti-mouse STAR Red (Sigma-Aldrich, 52283). Dilution 1:500.<br>Secondary goat anti-rabbit STAR Orange (Sigma-Aldrich, 41367). Dilution 1:500.<br>Secondary anti-mouse Alexa 568 (Thermo, A11004). Dilution 1:400.<br>Primary SCP-3 (D-1) antibody (Santa Cruz Biotechnology, SC-74569). Dilution 1:300.<br>Monoclonal ANTI-FLAG ® BioM2-Biotin (Sigma-Aldrich, F9291) conjugated to quantum dots (Thermo, Q21361MP). Dilution 1:66665. |
| Validation | Primary mouse anti-H3K27me antibody (Abcam, ab6002) validated for ChIP, ELISA, WB, IHC - Wholemount and ICC/IF applications.<br>Primary rabbit anti-H3K27ac antibody (Active Motif, 39034) validated by Active Motif for ChIP/ChIP-Seq, ICC/IF, WB and CUT&Tag applications.<br>Primary SCP-3 (D-1) antibody (Santa Cruz Biotechnology, SC-74569) validated for WB, IP, IF, IHC(P), FCM and ELISA applications. |

## Eukaryotic cell lines

Policy information about cell lines and Sex and Gender in Research

| | |
|---|---|
| Cell line source(s) | The cell lines used in this study were:<br>ATCC:CL-101<br>ATCC:CRL-2378.1 |
| Authentication | The following cell lines were not authenticated:<br>ATCC:CL-101<br>ATCC:CRL-2378.1 |
| Mycoplasma contamination | ATCC:CRL-2378.1 cells were confirmed to be free of mycoplasma by testing with the INTRON Mycoplasma PCR Detection Kit (#25234).<br>ATCC:CL-101 cells were not tested for mycoplasma contamination. |
| Commonly misidentified lines<br>(See ICLAC register) | ATCC:CRL-2378.1 - Parent cell line (MA-104) has been shown to be from African Green monkey instead of Rhesus macaque. |

## Animals and other research organisms

Policy information about studies involving animals; ARRIVE guidelines recommended for reporting animal research, and Sex and Gender in Research

| | |
|---|---|
| Laboratory animals | CD1 mature (10 to 12 weeks old) male mice were used. |
| Wild animals | This study did not involve wild animals. |
| Reporting on sex | Sex-based analyses were not relevant for this study. |
| Field-collected samples | This study did not involve samples collected from the field. |
| Ethics oversight | Animal and plant experimental procedures treated at the Instituto de Biotecnología (IBt) were approved by the Bioethics Committee of the Instituto de Biotecnología of the Universidad Nacional Autónoma de México (UNAM). Animal experimental procedures treated at the Department of Biomedicine and Prevention at Faculty of Medicine were approved by the "Ministero della Salute" of Italy, authorization n. 701/2018-PR. Animal experimental procedures treated on the Neurobiology and Epigenetics Unit of the European Molecular Biology Laboratory were approved by the EMBL Rome Animal Facility in accordance with European and Italian legislations. |

Note that full information on the approval of the study protocol must also be provided in the manuscript.
